# Variety of femoral anteversion and its measurement in cementless total hip arthroplasty: Does robotic technology improve accuracy?

**DOI:** 10.1186/s13018-024-04527-z

**Published:** 2024-01-09

**Authors:** Hongyi Shao, Yong Huang, Dejin Yang, Wang Deng, Xiang-Dong Wu, Yixin Zhou

**Affiliations:** Department of Orthopaedic Surgery, Beijing Jishuitan Hospital, Capital Medical University, Fourth Clinical College of Peking University, National Center for Orthopaedics, Beijing, 100035 China

**Keywords:** Femoral anteversion, Stem anteversion, Cementless total hip arthroplasty, Robotic-assisted THA

## Abstract

**Background:**

High-performance total hip arthroplasty (THA) depends on the accurate position of components. However, femoral anteversion is variable, and current studies only used traditional instruments to evaluate it, such as protractor and spirit level with limited cases. This study aimed to identify the variability in the measured femoral native anteversion and intraoperative stem anteversion under different measurement methods, including intraoperative robotic method. We hypothesized that robotic technology was more accurate than traditional instruments for femoral anteversion evaluation.

**Methods:**

This study included 117 hips of patients who underwent robotic-assisted THA between November 2019 and March 2021. Preoperative native femoral anteversion was measured using a robotic system. Intraoperative femoral stem anteversion was evaluated visually, and then measured with a goniometer and a robotic system, respectively. Variability in the measured femoral native anteversion and intraoperative femoral stem anteversion was calculated and compared. Intraclass correlation coefficient (ICC) and Pearson correlation analysis were used to assess the consistency and correlation of anteversion of different measurements and postoperative CT-measured stem anteversion, respectively.

**Results:**

The result of measurement for preoperative native femoral anteversion was more variable than the intraoperative robotic-measured stem anteversion. Intraoperative robotic-measured stem version showed the highest correlation with postoperative CT measurement of stem version (r = 0.806, *P* < 0.001), while intraoperative surgeon estimation had the lowest correlation coefficient (r = 0.281, *P* = 0.025). As for the consistency with postoperative CT measurement of femoral stem anteversion, the intraoperative robotic-measured femoral stem version also had the highest value (ICC = 0.892, *P* < 0.001).

**Conclusion:**

Native femoral anteversion was variable preoperatively. Using cementless stems, anteversion was also highly variable. Robotic assessment for stem anteversion during surgery was more consistent with the final position than the preoperative assessment and conventional intraoperative estimation.

## Introduction

Minimizing the dislocation rate after total hip arthroplasty (THA) largely depends on the accurate positioning of the components [1–3]. Suboptimal placement of the acetabular component or femoral stem may increase polyethylene wear, resulting in instability or even aseptic loosening [4–6]. Among the parameters of component position, anteversion is of great value because it could help avoid impingement during hip motion, which is required for activities in daily living [7]. Previously, Jolles et al. found that the dislocation rate was 6.9 times higher if the total anteversion (the sum of cup anteversion and stem anteversion) was outside of a range of 40°–60° [8]. With further understanding of the mechanism of spinopelvic motion, the problem of acetabular anteversion has been emphasized dynamically, not only in the standing position, but also in the sitting position [9, 10]. Tezuka et al. believed that hip dislocation was related to the combined sagittal index (CSI), which is the sum of ante-inclination and pelvic femoral angle [11]. However, CSI was related to acetabular anteversion, and pelvic femoral angle was related to hip flexion, while femoral anteversion was ignored.

Femoral anteversion is an important component of the concept of "combined version," which was initially proposed by Mckibbin [12]. Excessive femoral anteversion may induce posterior impingement and anterior dislocation of the hip when the hip extends. In contrast, inadequate femoral anteversion or retroversion may result in anterior impingement and posterior dislocation of the hip when the hip flexes [13]. Padgett emphasized the importance of femoral anteversion in THA stability, which cannot be fully compensated by acetabular anteversion [14]. This theory is further demonstrated using a mathematical model [15]. However, femoral anteversion is variable, and it was demonstrated by Reikeras et al. [16], in which the femoral version ranged from 17° of retroversion to 60° of anteversion using CT to measure anteversion. Park et al. studied the variation from preoperative CT to postoperative femoral anteversion and found that the difference was from 2.3° to 9.4° [17]. Hirata et al. even found that the results of femoral anteversion measured using a goniometer during surgery are different from those measured by postoperative CT scans [18]. Recently, the application of robotic-assisted THA has enabled surgeons to evaluate femoral anteversion during surgery accurately, which may be a potentially promising accurate technology [14]. However, few studies have reported on the variation of the femoral anteversion angle during robotic-assisted THA [19, 20]. Nodzo et al. assessed the accuracy of femoral anteversion using a robotic system, but the number of subjects included in this study was limited to 20 patients [21]. Until now, no studies have compared the accuracy of different evaluation methods for femoral anteversion during surgery, including robotic-assisted technology.

This study aimed to identify the variability in the measured femoral native anteversion (FNA) and intraoperative stem anteversion in our cohort of patients, and to answer whether robotic technology was more accurate than traditional instruments for evaluation of femoral anteversion. We hypothesized that there exists a wide range of FNA, and robotic-assisted THA would be a more accurate approach for the evaluation of stem anteversion.

## Material and methods

### Study design and participants

This study was approved by the institutional review board. Patients who underwent robotic-assisted THA between November 2019 and March 2021 were retrospectively included in this study. The exclusion criteria were intraoperative abortion of robotic surgery, intraoperative loosening of the screw anchoring the infrared trackers on the femoral side, and subtrochanteric or extended greater trochanter osteotomy of the femur. Finally, 117 hips of patients were included in our study. Among these, 64 hips had postoperative CT scans for other study programs to assess the position of acetabular components. Demographic data are summarized in Table 1.

**Table 1:** Patient demographics

Demographic	N = 117 Patients with different femoral version measurements	N = 64 Patients with postoperative CT
Age (years)*	52.36 ± 13.62 (20–87)	51.91 ± 12.91 (21–78)
Height (cm)*	166.34 ± 8.41 (150–180)	166.67 ± 8.91 (150–184)
Weight (kg)*	69.65 ± 14.01 (43–140)	69.59 ± 13.03 (43–100)
Body mass index (kg/m^2^)*	25.01 ± 3.52 (17.67–40.91)	24.90 ± 3.30 (18–32)
Gender (Male/Female ratio)	54 males: 63 females	29 males: 35 females
Side (Left/Right ratio)	55 left: 62 right	27 left: 37 right
DDH (%)	63 (53.8%)	35 (54.7%)

^*^Values are expressed as  (mean ± standard deviation) range

### Preoperative planning and measurement of femoral anteversion

All patients who underwent robotic-assisted THA (MAKO Stryker Inc., Fort Lauderdale, FL, USA) underwent a CT scan (Toshiba 320; 0.5 mm for pelvis and 2.0 mm for knee) for preoperative planning. Preoperative femoral anteversion was measured on the Mako workstation, which was the angle formed by the projection of the line connecting the femoral head center and femoral neck center with the line of the surgical epicondylar axis on the transverse plane when the patient's thigh was adjusted to parallel the longitudinal axis of the body.

### Surgical technique and intraoperative measurement of stem anteversion

**Fig. 1:**
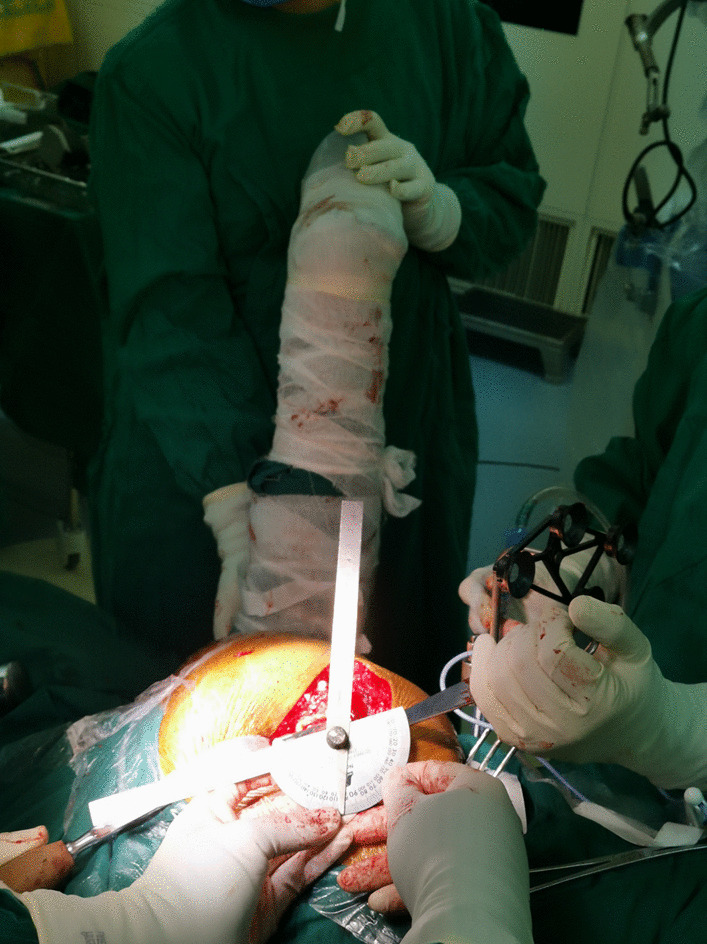
Intraoperative measurement of the stem anteversion using a goniometer

**Fig. 2:**
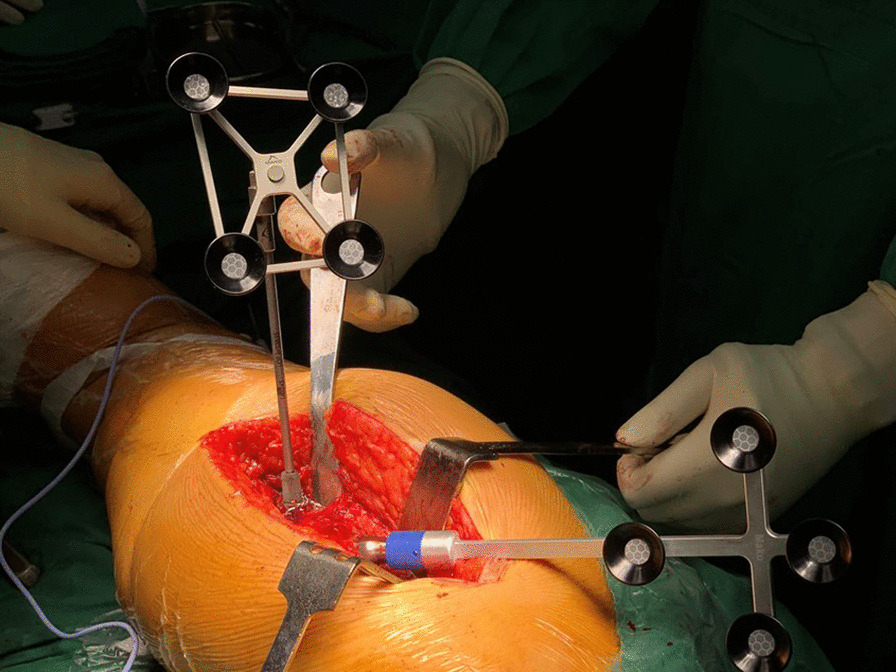
Intraoperative measurement of the stem anteversion using a robotic system

All surgical procedures were performed by three surgeons (Drs Yixin Zhou, Hongyi Shao, and Dejin Yang) with high levels of experience in hip arthroplasty. All of the included patients underwent THA with a posterior approach. First, we prepared the femoral side using the combined anteversion technique [22]. After inserting the trial into the femur, we tried to measure the femoral stem anteversion using three independent methods (visual assessment, goniometer, and robotic system) sequentially to figure out which method was more accurate and reliable. We then decided the acetabular anteversion to ream and implant cups using a robotic-assisted system. Finally, we implanted stems and measured stem anteversion using a goniometer. The angle was between the low limb and stem axis when flexing the knee and letting the tibia be vertically positioned (Fig. 1). Subsequently, we used the robotic system to measure femoral stem anteversion (Fig. 2).

### Radiographic measurement

Mimics software (version 20.0; Materialize, Leuven, Belgium) was used to reconstruct the postoperative CT scan image and perform radiographic measurements, which we regarded as "gold standard." Two doctors (Dr. Hongyi Shao, and Dr. Dejin Yang) measured the postoperative stem anteversion based on the definition proposed by Dorr et al. [23]. The angle is formed by the femoral neck axis and the femoral coronal plane. Femoral neck axis is defined as line connects femoral head center and center of femoral neck. While femoral coronal plane is defined by using the middle high point of the greater trochanter and the surgical epicondylar axis. The first 20 cases were measured by both of them, and the remaining cases were measured separately. One surgeon measured 20 randomly selected cases again after 4 weeks.

In 2020, Widmer reported that functional stem anteversion from 5° to 25° provided the largest combined target zones [3]. We classified patients into in-range anteversion (from 5° to 25°) and out-of-range (less than 5° or more than 25°) groups depending on anteversion measurement.

### Statistical analysis

Continuous data were expressed as mean and standard deviation, while categorical data were expressed as frequencies and percentages. The Chi-square test was used to compare categorical data. Standard deviation (SD) and coefficient of variation were used to represent the data variation. Intraclass correlation coefficient (ICC) was used to assess the consistency of anteversion of different measurements and postoperative CT-measured stem anteversion, which represents the value of difference, respectively. Pearson correlation analysis was used to assess the correlation of stem anteversion from different measurement methods, and postoperative CT-measured stem anteversion, which represents the relevance, respectively. *P* < 0.05 was considered statistically significant. SPSS 19.0 (SPSS Inc., Chicago, IL, USA) and GraphPad Prism 5 (GraphPad Software Inc., California, USA) were used to analyze the data.

## Results

**Fig. 3:**
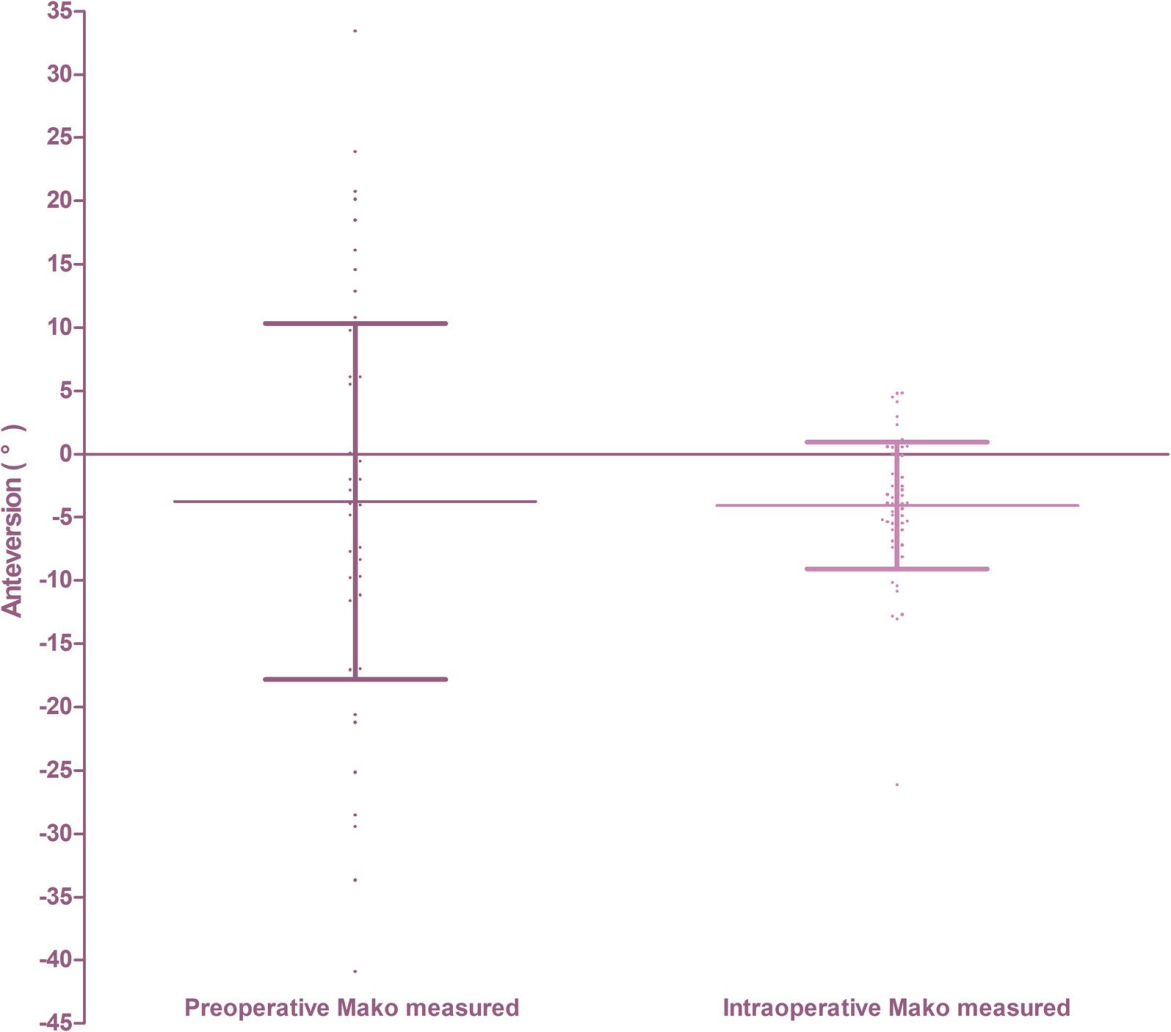
Variation in the preoperative robotic-measured native femoral anteversion and intraoperative robotic-measured stem anteversion. The scatter diagram of the preoperative robotic-measured femoral anteversion with more variation (mean: 13.85; SD: 14.49; coefficient of variation: 104.62%), and intraoperative robotic-measured stem anteversion with less variation (mean: 13.57: SD: 7.948; coefficient of variation: 58.57%)

All the 117 included patients were used the Mako robotic system to complete THA, including preoperative planning. All of them were implanted cementless cups (Trident, Stryker, Mahwah, NJ, USA). Among them, 11 patients received modular stems or conical stems (Table 2), while the other 106 patients received wedge-shaped cementless stems (Accolade II, Stryker, Mahwah, NJ, USA). The mean preoperative femoral anteversion measured by the robotic system was 13.8° ± 14.5° (range, − 36° to 50°; coefficient of variation, 104.62%), it was more variable than the intraoperative stem anteversion measured by the robotic system, which was 13.6° ± 7.9° (range, − 9° to 34°; coefficient of variation, 58.57%) (Fig. 3).

**Table 2:** Patients who received modular stem or conical stem to reconstruct femoral side

Case number	Reason for THA	TYPE OF STEM	Preoperative Mako-measured femoral version	Postoperative CT-measured femoral version
1	DDH	Wagner cone	11	20.67
2	DDH	S-ROM	32	26.47
3	OA	Wagner cone	− 4	29.68
4	DDH	Wagner cone	28	22.70
5	DDH	Wagner cone	28	21.32
6	DDH	Wagner cone	27	16.22
7	AVN	Wagner cone	45	24.86
8	DDH	Wagner cone	− 32	8.88
9	AVN	Wagner cone	19	Without CT
10	DDH	Wagner cone	21	Without CT
11	AVN	Wagner cone	38	Without CT

*DDH* development dysplasia of hip, *OA* osteoarthritis, *AVN* avascular necrosis

Among them, 63 cases have been diagnosed as developmental dysplasia of the hip (DDH), and the remaining 54 cases were non-DDH. Compared with non-DDH patients, DDH patients were more likely to have out-of-range preoperative anteversion (*P* = 0.028). However, after stem implantation, approximately one-fifth of the patients had out-of-range intraoperative stem anteversion in both DDH and non-DDH cases where after modular or conical stems were used (Table 3).

**Table 3:** Native femoral anteversion and intraoperative stem anteversion measured by robotic system for DDH and non-DDH patients

	Native femoral anteversion		Intraoperative stem anteversion measured by robotic system (with anteversion adjusted stem)		Intraoperative stem anteversion measured by robotic system (without anteversion adjusted stem)	
	< 5° or > 25°	5°–25°	< 5° or > 25°	5°–25°	< 5° or > 25°	5°–25°
DDH (n = 63)	33 (52.4%)	30 (47.6%)	11 (17.7%)	51 (82.3%)^**^	10 (18.2%)	45 (81.8%)
Non-DDH (n = 54)	17 (32.1%)	36 (67.9%)^*^	11 (20.4%)	43 (79.6%)	10 (19.0%)	40 (81.0%)
*P* Value	0.028		0.719		0.813	

^*^One case of traumatic arthritis was difficult to measure preoperatively

^**^One case of DDH lost Intraoperative Mako-measured femoral stem anteversion

**Fig. 4:**
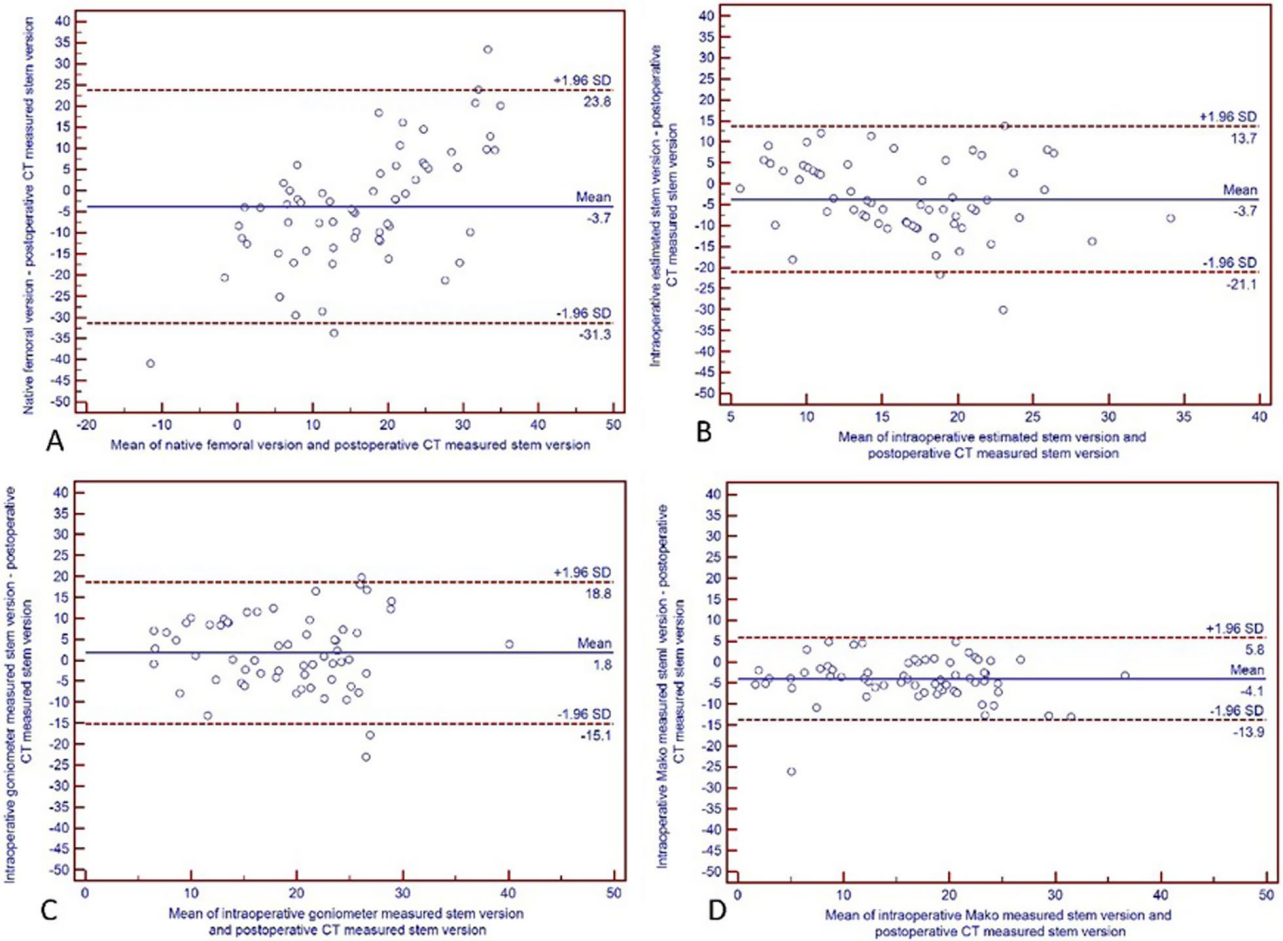
Consistency of femoral or stem anteversion of different measurements and stem anteversion postoperative CT measurement. Bland–Altman diagram demonstrating that the intraoperative robotic-measured stem version (**D**) was the most consistent with the postoperative CT-measured stem version when compared with the preoperative native femoral version (**A**), intraoperative surgeon estimated stem version (**B**), and intraoperative goniometer-measured stem version (**C**)

The intra-observer and inter-observer correlation coefficients of postoperative stem anteversion measurement were 0.995 and 0.986, respectively. Intraoperative robotic-measured stem anteversion showed the highest correlation with postoperative CT measurement of stem anteversion (r = 0.806, *P* < 0.001), while intraoperative surgeon estimation had the lowest correlation coefficient (r = 0.281, *P* = 0.025). Meanwhile, preoperative robotic measured native femoral anteversion (r = 0.435, *P* < 0.001) and intraoperative goniometer measured stem anteversion (r = 0.459, *P* < 0.001) had modest correlations (Table 4). As for the consistency with postoperative CT measurement of femoral stem anteversion, intraoperative robotic-measured femoral stem anteversion also had the highest value (ICC = 0.892, *P* < 0.001, Table 4, Fig. 4).

**Table 4:** Anteversion of different measurements and correlations with postoperative CT-measured stem anteversion

N = 64	Anteversion (Mean ± SD, range)	Postoperative CT-measured stem anteversion	r* (*P* value)	Intraclass correlation coefficient (*P* value)
Preoperative Mako-measured native femoral anteversion	14.64° ± 15.782° (− 32° to 50°)	18.37° ± 8.20° (3° to 38°)	0.459 (< 0.001)	0.546 (0.001)
Intraoperative surgeon estimated femoral stem anteversion	14.69° ± 6.41° (0° to 30°)		0.281 (0.025)	0.428 (0.014)
Intraoperative goniometer-measured femoral anteversion^**^	20.03° ± 7.96° (5° to 42°)		0.435 (< 0.001)	0.606 (< 0.001)
Intraoperative Mako-measured femoral stem anteversion	14.31° ± 7.87° (− 8° to 35°)		0.806 (< 0.001)	0.892 (< 0.001)

*Correlations between stem antevesion measured by postoperative CT and anteversion measured by other four methods. **Measurement of stem anteversion with goniometer after prosthesis implanted

## Discussion

The current study investigated variability in different measurement results of femoral anteversions and the relationship between different assessment methods of femoral and postoperative stem anteversions in our cohort of patients. We found that the measurement results of femoral anteversion and intraoperative stem anteversion were highly variable, which may compromise surgeons' ability to target functional combined safe zones. Among the different evaluation methods for stem anteversion, intraoperative measurement with the robotic system had a significant correlation and consistency with postoperative stem anteversion, which may help surgeons adjust the acetabular anteversion to achieve a specific safe zone intraoperatively.

Dorr et al. concluded that CSI, which combines acetabular and femoral positions in the sagittal plane, was a predictor of hip dislocation [10, 11]. However, they did not consider femoral anteversion, which influences femoroacetabular impingement of the hip joint [3]. Padgett reported that even though we should consider the relationship between the spine and hip for late dislocations, femoral stem anteversion was also important [14]. Increased femoral anteversion could induce posterior impingement in the standing position, while decreasing femoral anteversion could induce anterior impingement in the sitting position. Using the computerized 3-D model simulation technique, the findings were consistent with this trend [3]. Therefore, optimizing femoral anteversion is an important factor that should be considered. Since cementless stems are gaining popularity in clinical practice and their anteversion is difficult to adjust due to the dramatic change in the proximal femur morphology, surgeons usually prepare the femur first when they use the combined anteversion technique [22]. During our study, we found that preoperative femoral anteversion was highly variable, and nearly half of the patients had out-of-range femoral anteversion. Even after using modular or metaphyseal fixation stems to achieve optimal anteversion, approximately one-fifth of the patients remaining had out-of-range stem anteversion. This reaffirms the importance of preoperative and intraoperative measurements of femoral anteversion. Emerson et al. used MRI to compare postoperative stem anteversion and preoperative femoral anteversion and found that their results were inconsistent and variable [24]. They also postulated that the anatomic shape of the femoral canal determines the anteversion of the stem and highlights the importance of intraoperative measurement for stem anteversion.

Dysplastic hips may have a straighter diaphyseal canal, smaller neck shaft, and increased anteversion on the femoral side [25]. Wells et al. studied the femoral morphology of dysplastic hips based on CT scan data and found that the variety of femoral anteversion angles was very large [26]. They found that 8% of patients had relative femoral retroversion (< 5°), and 52% had excessive femoral anteversion (> 25°). Our findings are similar, and there were more dysplastic hips that had too large or too small femoral anteversion. After cementless stem insertion, more patients received in-range femoral anteversion, while nearly one-fifth were still outside the range. Femoral morphology, neck cutting level, and stem design all influence final stem anteversion, which makes it different from native femoral anteversion [27, 28]. Using a modular or conical stem to adjust anteversion and targeting in-range anteversion could explain why we obtained more optimal stem anteversion. However, cases of out-of-range anteversion should draw surgeons' attention, which may compromise clinical results.

In our study, we found that the preoperative femoral anteversion measured by the robotic system was not consistent with postoperative femoral stem anteversion. In the robotic system, preoperative femoral anteversion was defined by the angle of the projection of the line connecting the femoral head center with the femoral neck center and transverse epicondylar axis on the transverse plane. This measurement method and results were similar to those of a previous study that reported preoperative femoral anteversion from a 3-D CT scan, which was a little less than the postoperative results [17]. Although the postoperative femoral anteversion measurement method for the femoral component was the same as preoperative, the component position was related to the femoral morphology, the types of the femoral stem we used, and the surgical technique[23, 24, 29]. Thus, we suppose that the measurement of preoperative anteversion of the femur from the robotic system does not provide us with consistent intraoperative anteversion.

Both robotic systems and traditional instruments for measurements of intraoperative femoral anteversion provide more accurate results than preoperative estimation. The robotic measurements were more consistent with the real results. All THAs were performed using the posterior approach, and osseous landmarks were limited. Dorr et al. first reported the error of surgeon's estimation for femoral anteversion by as much as 11°, which was similar to our results [23]. Hirata et al. further reported that surgeons tended to overestimate femoral anteversion when they used the posterior approach, which was related to the severity of knee osteoarthritis [18]. Our results are consistent with this trend. Our study found that the robotic technique had highest accuracy for intraoperative femoral anteversion evaluation. Nodzo et al. used a robot to assist hip arthroplasty and found that the femoral anteversion measured during the operation was highly consistent with that measured by postoperative CT [21]. However, in three of the 20 hips, there was a difference of more than 5° between the intraoperative and postoperative CT measurements. The error of intraoperative registration and potential displacement of the markers may be the causes of bias. Nevertheless, the robotic system is still a reliable and accurate method that helps determine femoral anteversion intraoperatively. Because postoperative CT measurements, as the gold standard, cannot provide real-time intraoperative data. Using different intraoperative measurement methods to assist joint surgeons in obtaining estimates of intraoperative femoral anteversion can help them to perform surgeries more accurately.

There are several limitations to our study. First, this was a retrospective study, which has inherent limitations for the method itself. However, it was difficult do such kind of study prospectively and blindly. Second, the surgical technique and conceptions may influence stem anteversion. However, all surgeons who performed these surgeries were well-trained in our hospital, and their targets and approaches were the same. Third, only half of the patients in this study had postoperative CT data for analysis. Since CT had more radiation than X-ray, it was difficult to perform a CT scan for every patient after surgery, and the demographic data of patients who underwent CT were similar to those of patients who did not undergo CT. Finally, femoral stem anteversion assessment by visual and goniometer was relatively subjective, especially after the robotic system measured the stem trial. However, we routinely measured stem anteversion before we used the robotic system as previously described. Further prospective studies with more cases for femoral anteversion are warranted to confirm our findings and address these limitations.

## Conclusion

In conclusion, native femoral anteversion was variable preoperatively. With the use of cementless stems, anteversion was also highly variable. Robotic assessment for stem anteversion during surgery was more consistent with the final position than preoperative assessment and conventional intraoperative estimation, while the visual assessment was inaccurate.
